# New pneumococcal serotype 20C is a WciG O-acetyltransferase deficient variant of canonical serotype 20B

**DOI:** 10.1128/spectrum.02443-24

**Published:** 2024-11-29

**Authors:** Jigui Yu, Neil Ravenscroft, Peter Davey, Roshan Liyanage, Oliver Lorenz, Michelle M. Kuttel, Stephanie W. Lo, Feroze A. Ganaie, Moon H. Nahm

**Affiliations:** 1 Department of Medicine, Division of Pulmonary/Allergy/Critical Care, University of Alabama at Birmingham, Birmingham, Alabama, USA; 2 Department of Chemistry, University of Cape Town, Rondebosch, South Africa; 3 Vaxcyte, San Carlos, California, USA; 4 Parasites and Microbes, Wellcome Sanger Institute, Hinxton, Cambridge, United Kingdom; 5 Department of Computer Science, University of Cape Town, Rondebosch, South Africa; 6 Milner Centre for Evolution, Department of Life Sciences, University of Bath, Bath, United Kingdom; Emory University School of Medicine, Atlanta, Georgia, USA

**Keywords:** *Streptococcus pneumoniae*, capsule polysaccharide, serogroup 20, O-acetyltransferase, vaccine

## Abstract

The polysaccharide (PS) capsule of *Streptococcus pneumoniae* (pneumococcus) is the immunodominant surface structure that shields the bacteria from the host immune system. Since the capsule is the primary target of currently available pneumococcal vaccines, anti-capsular antibodies are highly protective but serotype-specific. Pneumococci may evade host or vaccine-induced immunity as a result of variation in capsule structure mediated via multiple mechanisms, such as the loss or gain of O-acetylation. Previous biochemical studies of serogroup 20 isolates have identified two subtypes—20A and 20B, whose capsule PS differs in the WhaF-mediated glucose side chain. Herein, we characterize a newly discovered capsule type, 20C, that differs from serotype 20B via the inactivation of capsule O-acetyltransferase gene, *wciG*. Structural analysis demonstrated that 20C and 20B share an identical repeat unit [→3)-α-D-Glc*p*NAc-[β-D-Gal*f*-(1→4)][α-D-Glc*p*-(1→6)]-(1→P→6)-α-D-Glc*p*-(1→6)- β-D-Glc*p*-(1→3)-β-D-Gal*f* 5,6Ac_2_-(1→3)-β-D-Glc*p*-(1→], except for the absence of WciG-mediated O*-*acetyl group at terminal galactofuranose (β-D-Gal*f*). We confirmed that deletion of the *wciG* gene in a 20B strain resulted in the expression of the 20C capsule. Serotype 20C is serologically indistinguishable from the canonical 20A and 20B using conventional serotyping antibodies, but serogroup 20 subtypes can be distinguished by sequencing of *cps* genes—*whaF*, *wciG*, and *wcjE*. While genetic screening suggests 20C to be globally less prevalent, a new variant was identified which appears to have both *wciG* and *whaF* genes inactive, potentially indicating it to be a new serotype. Consequently, genome-based serotyping/bioinformatic tools must scrutinize all *cps* genes for mutations that might inactivate/modify *cps*-encoded enzymes, ensuring effective tracking of emerging capsule variants in response to ongoing vaccination efforts.

**IMPORTANCE:**

*Streptococcus pneumoniae* (pneumococcus) is a significant human pathogen known for producing a wide array of antigenically and structurally diverse capsule types, a fact that poses a serious challenge to the effectiveness of vaccines targeting pneumococcal capsule polysaccharide (PS). Herein, we provide a comprehensive analysis-genetic, antigenic, and biochemical of a newly identified capsule type, 20C, which differs from the canonical serotype 20B due to the inactivation of the capsule O-acetyltransferase gene, *wciG*. Our findings highlight how pneumococci can alter their capsule PS structure and immunological characteristics through minor genetic modifications. Since the appearance of new capsule types can directly affect pneumococcal conjugate vaccine (PCV) implementation, a deeper understanding of capsule PS at the genetic, immunological, and biochemical levels is critical for the development of future diagnostic tools and vaccines.

## INTRODUCTION

*Streptococcus pneumoniae* (the pneumococcus) is a major human respiratory tract pathogen that typically colonizes the nasopharynx but can also cause a wide range of invasive and non-invasive diseases (1). Despite the availability of vaccines and antibiotics, pneumococcal infections remain a significant cause of morbidity and mortality accounting for ∼9 million cases of infection per year with ∼300,000 lethal cases (2, 3). Its survival in human hosts is facilitated by the production of polysaccharide (PS) capsule—a major immunogen that plays a critical role in virulence, principally by interfering with host opsonophagocytic clearance mechanisms (4, 5). As a species, pneumococcus is capable of producing over 100 antigenically and biochemically unique capsule types (serotypes) (6–9), and the protection provided by the anti-capsule antibodies is generally serotype-specific.

The capsule is an active component of the currently available pneumococcal conjugate vaccines (PCVs), and the highly successful vaccination strategies are designed to elicit antibodies to the serotypes causing high rates of invasive pneumococcal disease (IPD) (10). Though widespread vaccination efforts have been successful in reducing the incidence of IPD in children and adults (11, 12), infections caused by non-vaccine types have subsequently increased (13). Since only a limited number of serotypes are targeted by the vaccines, non-vaccine serotypes can occupy the ecological niche left by the overall reduction of vaccine-targeted serotypes (14, 15). As changes in the pneumococcal population are expected in response to temporal variation or host/vaccine-induced selective pressure, it is important to continuously survey the diversity of pneumococcal serotypes (16). Monitoring the capsule variability among vaccine-related serogroups is essential, as existing vaccines may not provide cross-protection against certain structurally related non-vaccine serotypes (17, 18).

**Fig 1:**
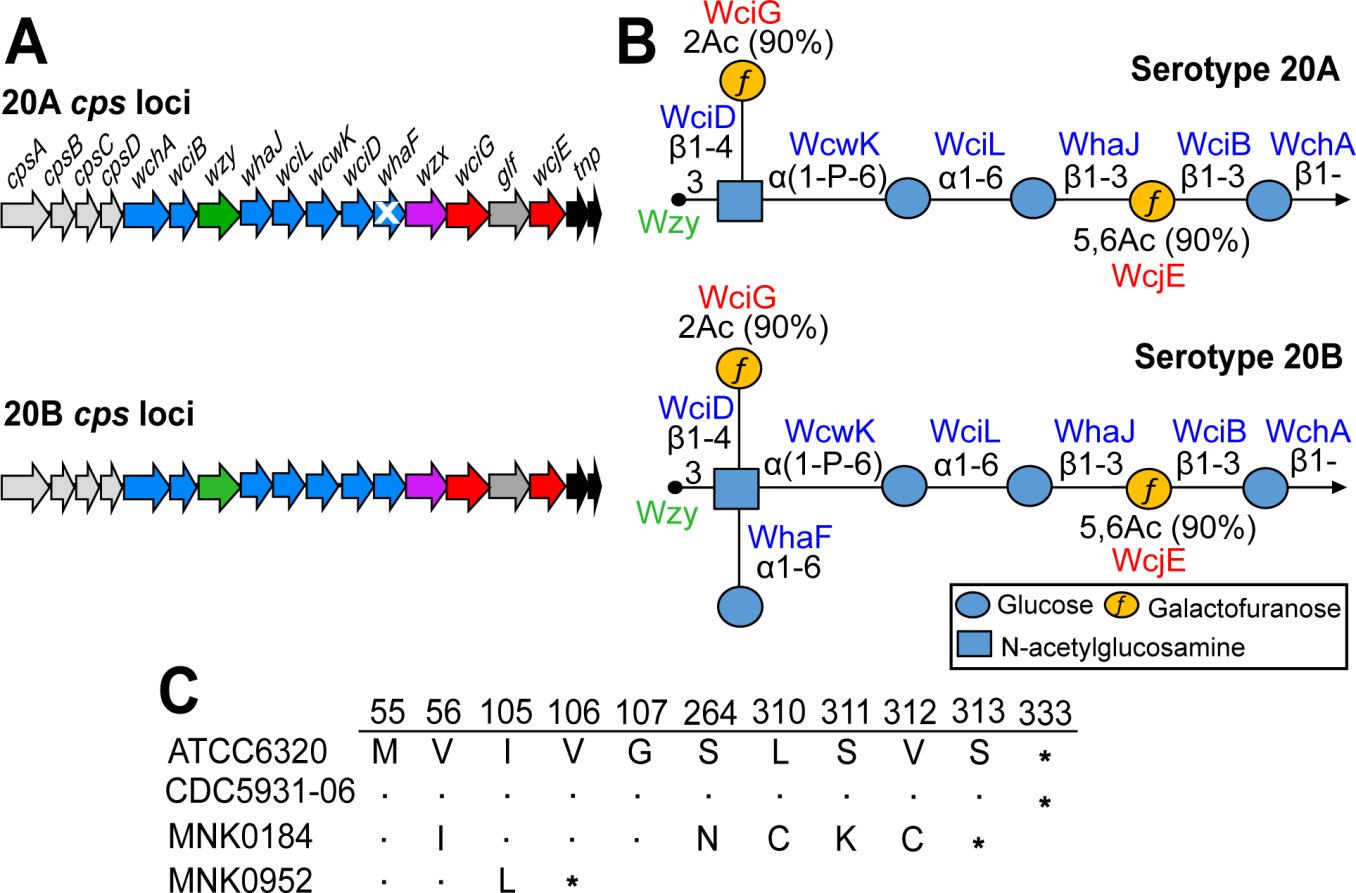
Capsule synthesis loci and capsule polysaccharide structures of serogroup 20 subtypes. (**A**) Representative *cps* loci of serotypes 20A and 20B. Highly conserved regulatory genes (light gray arrows), genes encoding glycosyltransferases (blue arrows), Wzy polymerases (green arrows), Wzx flippases (pink arrows), O-acetyltransferases (red arrows), carbohydrate synthetases (dark gray arrows), transposable elements (black arrows) are labeled at the top of 20A *cps* locus. Same gene annotation is followed for the 20B *cps* locus. White color cross on the *whaF* gene arrow in the 20A *cps* locus indicates that this gene is defective. *cps*, capsule synthesis locus. (**B**) Symbol nomenclature for glycans diagrams of elucidated serotypes 20A and 20B PS repeat unit structures. *cps*-encoded glycosyltransferase, O-acetyltransferases, and polymerase are listed in blue, red, and green text, respectively, on top of their putatively assigned linkages. (**C**) Alignment of WciG amino acid sequences showing substitutions and translational defects at different residues. The alignment shows WciG sequences of serotype 20A (ATCC6320), serotype 20B (CDC5931-06), and WciG variants (MNK0184 and MNK0952). The numbers on the top of the alignment refer to the amino acid position. Symbol (*) indicates the stop codon. Dots refer to conserved amino acids in reference to the ATCC6320 WciG sequence.

Serotype 20 used to be an “orphan” serotype (i.e., lacked other serologically related serotype) until serologic studies of serotype 20 isolates led to the discovery of two serologic subtypes—20A and 20B (19, 20). Biochemical studies demonstrated that subtype 20A produced a hexasaccharide repeat unit (RU) (20), whereas subtype 20B produced a heptasaccharide RU containing an extra glucose side chain attached to the previously described structure (20, 21). The structural difference was attributed to the glucosyl transferase gene (*whaF*), which is intact in 20B but defective in 20A (20) (Fig. 1A and B). Until recently, serotype 20A was included solely in the 23-valent polysaccharide vaccine (PPSV23), but it is now incorporated in the 21-valent PCV for adults (22, 23) that has been approved by the U.S. FDA in 2024 (24). Albeit in a low proportion, serotype 20 isolates have been found in the nasopharynx of children (25–27) and reported as a cause of IPD (28–30). Despite limited knowledge about their specific virulence characteristics, serotype 20 isolates have been linked to increased disease severity, invasiveness, and mortality (30, 31).

The limited epidemiological data on serogroup 20 isolates suggests that serotype 20B remains the predominant serogroup 20 subtype worldwide (20). Recently, a 20B variant was identified that produces 20B capsule without O-acetylation on terminal galactofuranose (tGal*f*) (32). Herein, we determined that the 20B variant is a new serotype, named serotype 20C by confirming its unique capsule PS structure and demonstrating its genetic basis as a defective O-acetyltransferase gene, *wciG*.

## RESULTS

### Multiple serogroup 20 isolates contain variably inactive O-acetyltransferase WciG

We examined 20 pneumococcal isolates previously typed as serotype 20 using conventional serological reagents (data not shown). The bacterial panel included strains ATCC6320 and CDC5931-06, representing serotypes 20A and 20B, respectively (20). The *cps* loci of reference 20A strain, ATCC6320 (GenBank accession: JQ653094), and 20B strain, CDC5931-06 (GenBank accession: JQ653093) are syntenic and exhibit 99.9% nucleotide identity (*cpsA-glf*), with both harboring an intact, 999 bp (333 amino acids), O-acetyltransferase gene, *wciG* (Fig. 1A). Analysis of the *wciG* gene across all serotype 20 isolates revealed sequences identical to the reference 20B strain CDC5931-06, except for MNK0184 (GenBank accession: PQ205320) and MNK0952 (GenBank accession: PQ205321). As depicted in Fig. 1C, the WciG of MNK0184 exhibited several amino acid changes after residue 310 and lost 20 amino acids at the C-terminus. Similarly, the WciG of MNK0952 is truncated, terminating at residue 106. These variable nonsense mutations should result in premature translation termination and may render WciG non-functional in both strains. The rest of their *cps* locus was intact and identical to the reference 20B *cps* locus (JQ653093). Consequently, we hypothesized that these WciG variants are likely to produce a unique capsule PS distinct from canonical 20A and 20B.

### Serogroup 20 WciG variants produce capsule PS distinct from canonical 20A and 20B, but identical to the 20B variant

To examine our hypothesis, we purified and biochemically analyzed capsule PS from the WciG variants of serogroup 20 strains, MNK0184 and MNK0952. Glycosyl composition analysis of trifluoroacetic acid-treated samples detected N-acetylglucosamine (GlcNAc), galactose (Gal), and glucose (Glc) in approximately 1:2:4 ratio (Table 1), unlike 20A, which is known to have a hexasaccharide RU with 1:2:3 ratio (20). The findings suggested that MNK0184 and MNK0952 produce capsule PS with heptasaccharide RU as described for serotype 20B (20).

**TABLE 1:** Glycosyl composition analysis of purified capsule polysaccharide^*a*^

Sample name	Concentration (mM)^*b*^			Molar ratio		
	GlcNAc	Gal	Glc	GlcNAc	Gal	Glc
CDC5931-06	0.8045	1.3342	3.2898	1	2	4
MNK0184	1.2662	2.0691	5.0036	1	2	4
JY21	0.9168	1.4929	3.7144	1	2	4

^*a*^ HPAEC-PAD analysis revealing carbohydrate composition of trifluoroacetic acid-treated capsule PS purified from pneumococcal strains, CDC5931-06 (serotype 20B), MNK0184 (serotype 20C), and JY21 (*wciG*-knock out of CDC5931-06). Glc, Glucose; Gal, Galactose; GlcNAc, N-acetylglucosamine.

^*b*^ Concentrations shown here are one representative data set from three replicates.

**Fig 2:**
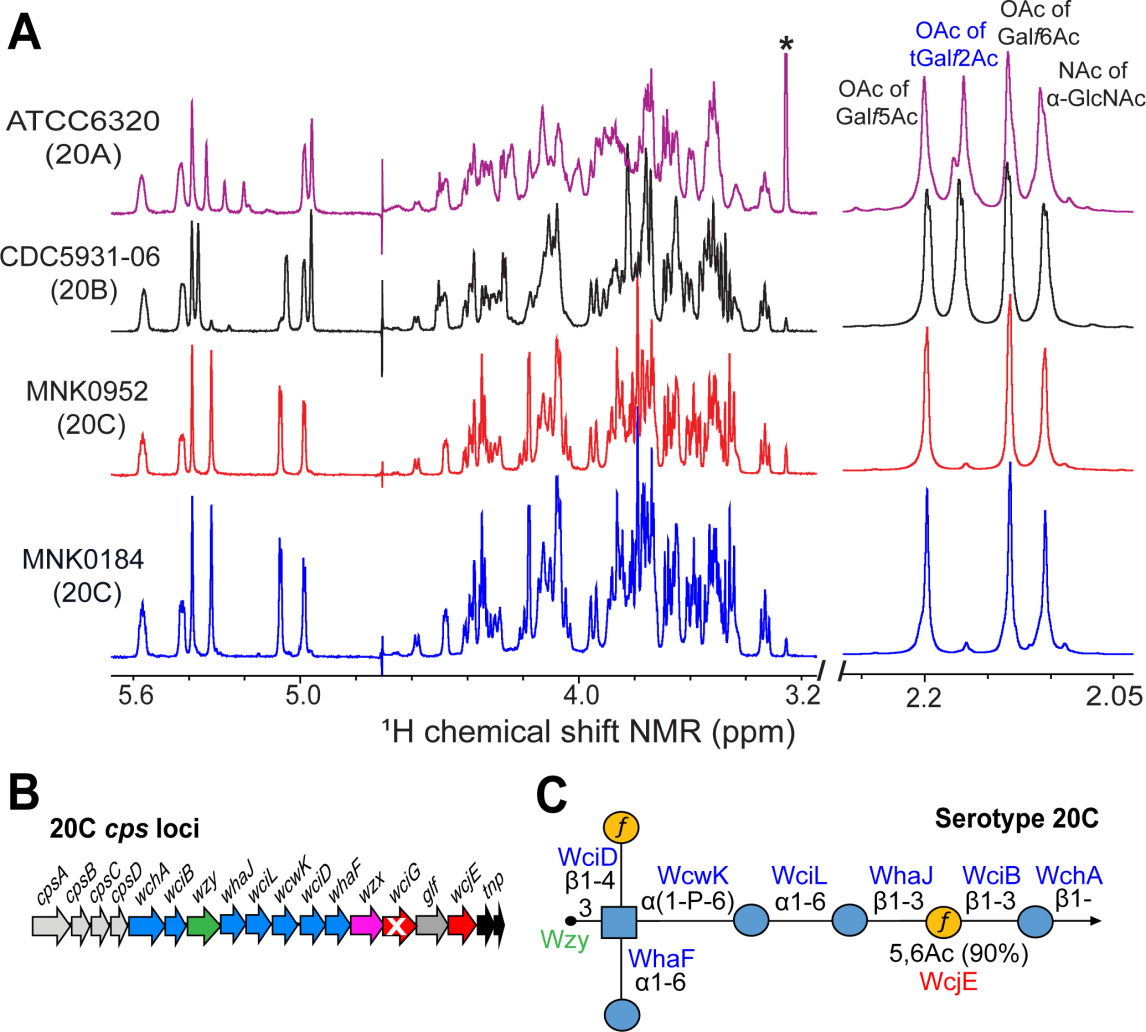
Biochemical analysis of capsule polysaccharide produced by serogroup 20 WciG variants. (**A**) ^1^H NMR spectra of native capsule PS purified from strains of serotype 20A (ATCC6320), 20B (CDC5931-06), 20C (MNK0184 and MNK0952). Acetylation peaks in the O-acetyl methyl region are labeled (chemical shift: 2.05–2.25 ppm), and O-acetylation of terminal galactofuranose (tGal*f*) is labeled in blue letters. Asterisk denotes a signal arising from the cell wall polysaccharide (CWPS). Strain name is provided on the left of the spectra and the corresponding serotype is in parenthesis. (**B**) *cps* locus of serotypes 20C. The gene annotation is followed as described for 20A and 20B *cps* loci in Fig. 1A. White color cross on the *wciG* gene arrow in the 20C *cps* locus indicates that this gene is defective. (**C**) Symbol nomenclature for glycans diagrams of elucidated serotype 20C PS repeat unit structure. *cps*-encoded glycosyltransferase, O-acetyltransferases, and polymerase are listed in blue, red, and green text, respectively, on top of their putatively assigned linkages. The symbol key is found in Fig. 1B.

Furthermore, we evaluated the capsule PS structures by nuclear magnetic resonance (NMR) and analyzed the diagnostic acetyl and anomeric regions. For direct comparison, purified capsule PS from canonical strains ATCC6320 (20A) and CDC5931-06 (20B) were also analyzed. The ^1^H NMR spectra of MNK0184 and MNK0952 are identical to each other and clearly shows the absence of major O-acetylation (OAc) peak at 2.17 ppm in the O-acetyl methyl region (chemical shift: 2.05–2.25 ppm), which otherwise are present in the canonical 20A and 20B capsule PS (Fig. 2A). This specific resonance signal corresponds to the O-acetyl group attached to the 2nd carbon of the terminal galactofuranose (tGal*f*) branch (32). Unlike the spectrum of CDC5931-06, which exhibits seven major peaks in the anomeric region between 4.8 and 5.6 ppm, the spectra of MNK0184/MNK0952 show only six (Fig. 2A). The lack of OAc on tGal*f* results in a slight upfield shift (0.05 ppm) of the H1 tGal*f* peak from 5.36 to 5.31 ppm and no resonance signal for H2 tGal*f* at 4.95 ppm. All other acetylation (OAc and NAc) peaks remain unchanged across all capsule PS. Furthermore, two-dimensional NMR data of MNK0184 and MNK0952 were completely identical to each other, thus, re-confirming their uniqueness as established by ^1^H NMR (Fig. S1) (32). In summary, the serogroup 20 WciG variants—MNK0184 and MNK0952 produce identical capsule PS but are distinct from the canonical 20A and 20B, thus representing a new serotype, named 20C (Fig. 2B and C).

### Inactivation of O-acetyltransferase, WciG, converts serotype 20B to serotype 20C

**Fig 3:**
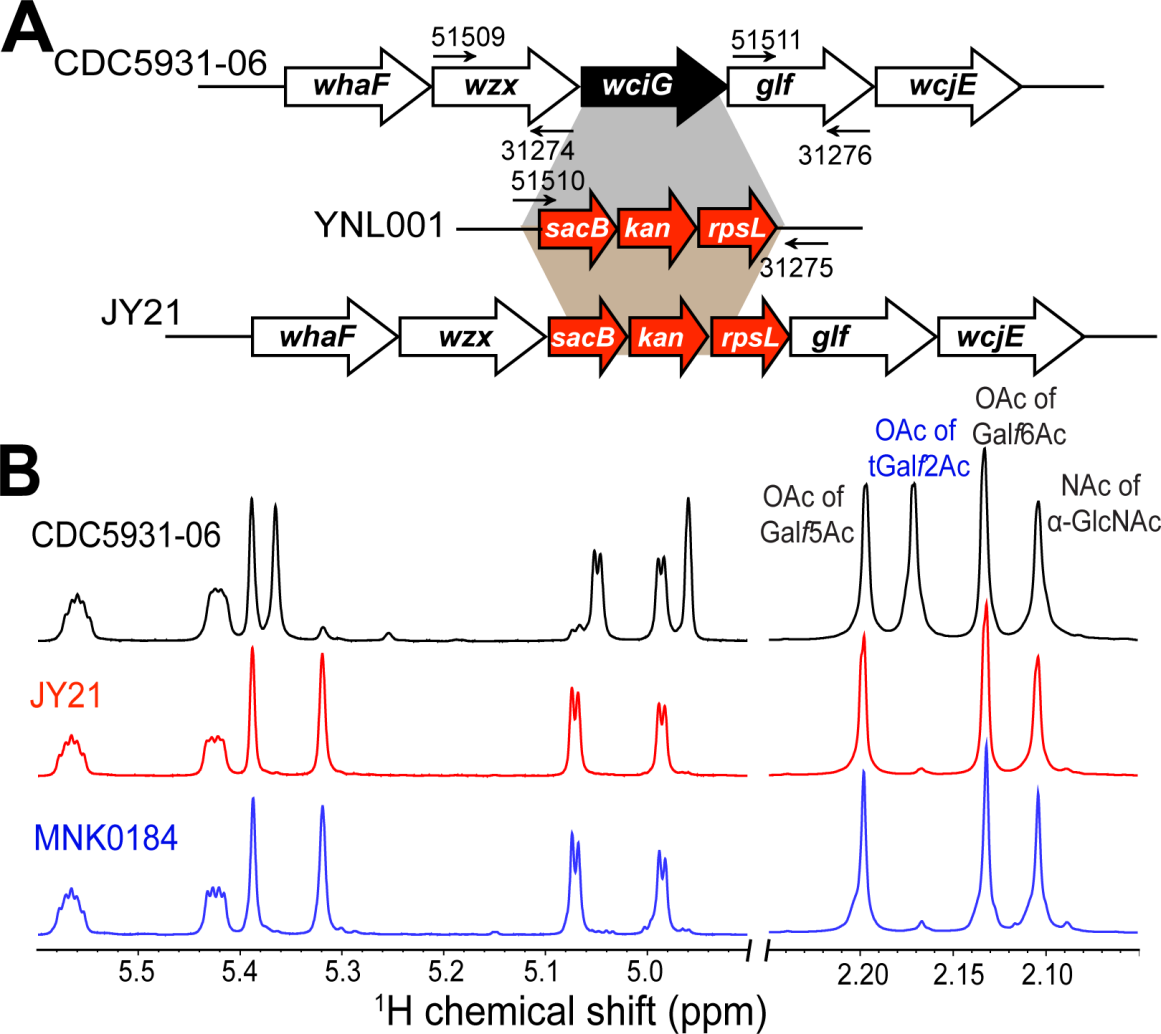
Deletion of *wciG* results in the loss of O-acetylation at the terminal galactofuranose (tGal*f*) branch of serotype 20B PS. (**A**) SJC strategy to create the *wciG* knock-out mutant strain, JY21, from CDC5931-06 (serotype 20B). YNL001 is a pneumococcal strain containing SJC (genes—*sacB*, *kan*, *rpsL*) (8, 34). The five-digit numbers indicate the PCR primers, which are described in Table S4. The black arrows (short and thin) underneath the primers indicate the direction of the primers. (**B**) ^1^H NMR spectra of native capsule PS purified from CDC5931-06 (serotype 20B, black spectra), JY21 (*wciG* knock-out, red spectra), and MNK0184 (serotype 20C, blue spectra). The ^1^H NMR overlay shows the signals arising in the anomeric (4.9 to 5.6 ppm) and acetyl-methyl (2.05 to 2.25 ppm) regions. Acetylation peaks are labeled, and the O-acetylation of tGal*f* is labeled in blue.

To verify that WciG non-functionality was responsible for the novel capsule type, 20C (Fig. 2B and C), we created a *wciG*-deficient recombinant mutant (JY21) of serotype 20B strain, CDC5931-06, using the Sweet-Janus Cassette (SJC) strategy (Fig. 3A) (7, 33). The ^1^H NMR spectrum of the native capsule PS purified from JY21 no longer contained the signal (2.17 ppm) corresponding to OAc of tGal*f* and was identical to that of MNK0184 (Fig. 3B). Furthermore, the monosaccharide composition of JY21 capsule PS was also identical to that of MNK0184 (Table 1). Altogether, the findings confirm that inactivation of O-acetyltransferase WciG in a serotype 20B strain results in the expression of the 20C capsule type.

### Serotype 20C cannot be distinguished serologically from canonical 20A and 20B and is effectively opsonized by anti-20A PS antibodies

**Fig 4:**
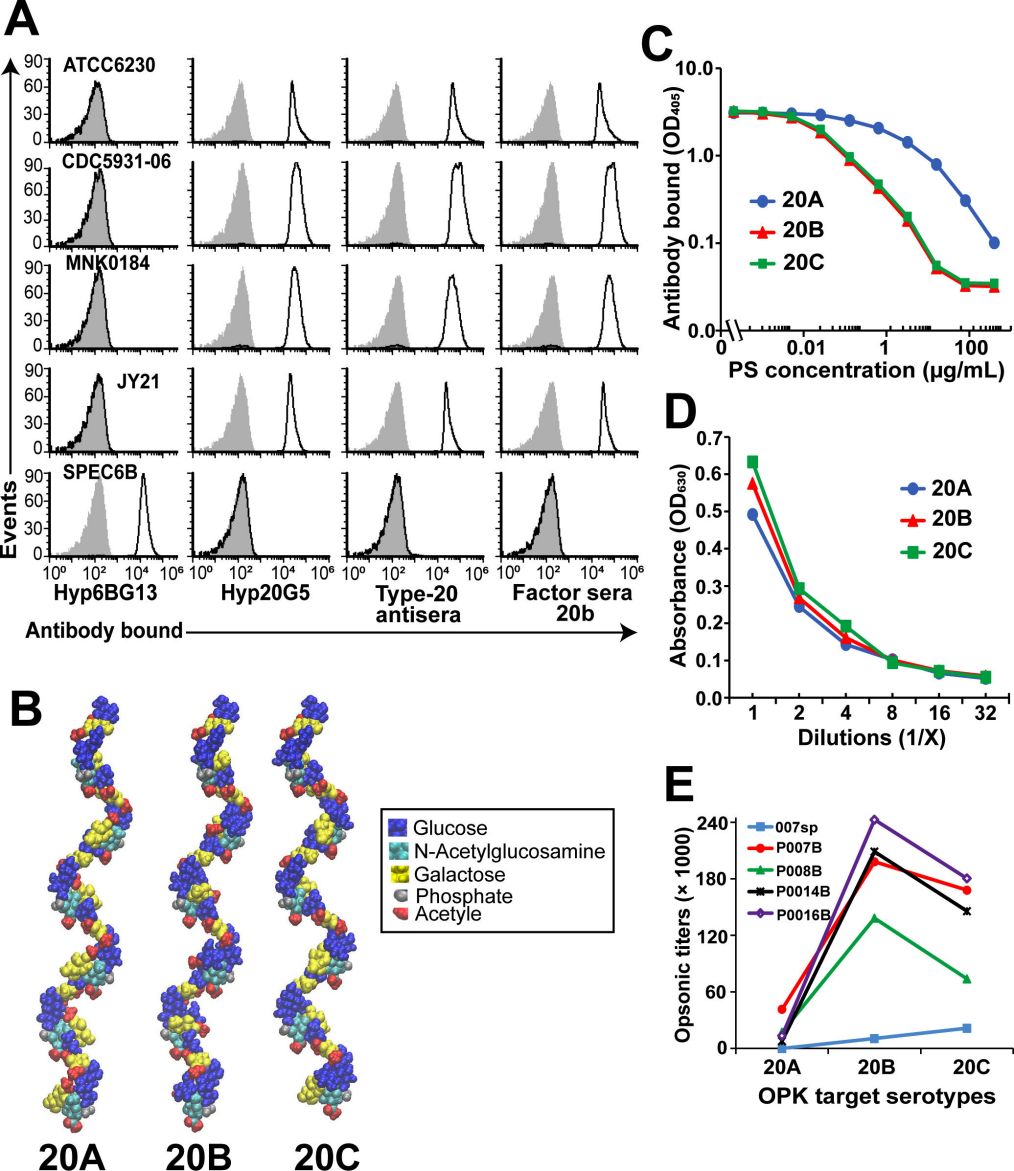
Serological properties of serogroup 20 subtypes. (**A**) FCSA histograms depicting antibody deposition on strains ATCC6320 (20A), CDC5931-06 (20B), MNK0184 (20C), JY21 (20C), and SPEC6B (serotype 6B, control strain). Black curves represent the fluorescence of bacteria incubated in different antisera (bottom labels), while gray-filled curves represent negative-control preparations incubated with secondary antibodies only. Hyp6BG13 and Hyp20G5 represent monoclonal antibodies (Mabs) specific to serotype 6B and serogroup 20, respectively. Type-20 antiserum and factor serum 20b are the polyclonal rabbit antisera. (**B**) Minimized molecular models for 6RU of 20A (left), 20B (middle), and 20C (right), shown in space-filling representation and colored according to residue type. The models show the same extended helical conformation with 2RU per helical turn for the three serotypes. (**C**) Inhibition enzyme-linked immunosorbent assay (ELISA) illustrates the ability of purified 20A, 20B, and 20C capsule PS to inhibit the binding of Hyp20G5. 20A capsule PS was commercially obtained from ATCC. 20B and 20C capsule PS were purified from CDC5931-06 and MNK0184 strains, respectively. The Y-axis shows the antibody (Hyp20G5) bound to ELISA plates coated with 20A PS, while the X-axis displays different inhibitory concentrations of purified capsule PS. (**D**) Reactivity of purified 20A, 20B, and 20C capsule PS with the anthrone reagent. Y-axis represents the absorbance at OD_630_, indicating the strength of reactivity, and X-axis shows different PS dilutions (twofold serial dilutions) used to react with the anthrone reagent. Average OD values from quadruplets are plotted. (**E**) Opsonic titers against target strains ATCC6320 (20A), CDC5931-06 (20B), MNK0184 (20C) using postimmunization PPSV23 sera samples from four human adults (P007B, P008B, P014B, and P016B) and a reference sample (007sp). Data were analyzed by one-way analysis of variance with Tukey’s multiple-comparison test. OPK, opsonophagocytosis killing.

To investigate if serotype 20C could be serologically distinguished from serotypes 20A or 20B, we tested two commercially available rabbit antisera*,* i.e.*,* type 20 antiserum and factor sera 20b, and a type 20-specific monoclonal antibody (Mab), Hyp20G5, for binding to serotype 20C (MNK0184 and JY21), 20A (ATCC6320), and 20B (CDC5931-06) isolates in a flow cytometric serotyping assay (FCSA). Consistent with all serogroup 20 subtypes being previously typed as serotype 20 (20), ATCC6320, CDC5931-06, MNK0184, and JY21 showed strong reactivity with all the antisera tested (Fig. 4A). Mean fluorescence intensity indicated that all the antisera bound with comparable efficacy, showing no appreciable serological difference. Thus, serogroup 20 subtypes—20A, 20B, and 20C are indistinguishable using the currently available serotyping antibodies. The observed serological cross-reactivity within serogroup 20 subtypes is consistent with the molecular modeling studies which suggest that the 20A, 20B, and 20C PS all exhibit the same extended helical conformation with 2RU per helical turn (Fig. 4B).

Next, we evaluated the efficiency of purified 20A, 20B, and 20C capsule PS to inhibit the binding of Hyp20G5 in an inhibition enzyme-linked immunosorbent assay (ELISA). We identified that 20B and 20C capsule PS inhibited Hyp20G5 binding ~100-fold better than 20A PS, although 20B could not be distinguished from 20C (Fig. 4C). To ensure that the observed inhibition was mediated by the equivalent amounts of capsule PS, we subjected the same samples used in the inhibition ELISA to a colorimetric “anthrone reactivity test” (35). This test detects and semi-quantifies carbohydrates in an acidic solution. As illustrated in Fig. 4D, the capsule PS from all three serotypes (20A, 20B, and 20C) reacted equally well with the anthrone reagent across various PS dilutions, thus eliminating any ambiguity regarding the quantity of PS used in the inhibition ELISA.

Furthermore, to explore the possibility of cross-protection among the serogroup 20 subtypes, we investigated whether immunization with the PPSV23 containing 20A PS induces cross-reactive, functional antibodies to 20B and 20C in human adults. Postimmunization serum samples from five anonymous human individuals were tested against ATCC6320, CDC5931-06, and MNK0184 targets in an *in vitro* opsonophagocytosis killing (OPK) assay (Fig. 4E). One of the samples tested was 007sp, which is the reference serum from FDA and was prepared by pooling sera from vaccinated adults (36, 37). Despite being immunized with 20A PS, the immune sera tended to opsonize serotypes 20B and 20C more effectively (sixfold to eightfold higher) than serotype 20A (Fig. 4E). The opsonic titers for serotype 20B (mean = 1.6 × 10^5^, *P* = 0.004) and serotype 20C (mean = 1.18 × 10^5^, *P* = 0.0269) were significantly higher than for serotype 20A (mean = 1.9 × 10^4^), with 20B exhibiting a marginally higher killing titer compared to 20C (*P* = 0.3920). While immunological studies with additional samples should be performed in the future, it is likely that 20A PS would elicit protective antibodies against 20B and the newly identified serotype, 20C.

### Serotype 20C may have a low global prevalence and potential emergence of new serogroup 20 variants

In addition to the pneumococcal clinical isolates from the University of Alabama at Birmingham (UAB) bacterial repository (*n* = 20) (Table S1), a comprehensive analysis of pneumococcal genomes from the Global Pneumococcal Sequencing (GPS) database (*n* = 21,199) identified 35 serogroup 20 isolates with whole-genome sequencing (WGS) data (Table S2). Based on the intactness of the *whaF* gene, there were 23 presumptive serotype 20A isolates and 11 serotype 20B isolates. One carriage isolate (GPS_US_PATH4317; accession number: ERR1453805), herein referred to as “PATH4317” from Ethiopia belonged to Global Pneumococcal Sequence Cluster 667 and exhibited sequence defects in both *wciG* and *whaF* genes, marking a novel genetic feature among serogroup 20 isolates (Fig. S2A). Specifically, the *wciG* gene harbored a 163 bp insertion between nucleotides 301 and 465, resulting in premature translation termination at amino acid residue 107. The *whaF* gene had a substantial deletion of 714 bp in the middle, resulting in the loss of 238 amino acids between residues 42 and 281. Consequently, the resulting WhaF polypeptide is significantly shorter (83 amino acids instead of 321) and likely non-functional. The fact that PATH4317 *cps* locus displays a unique gene content, it is likely that it may produce a capsule PS distinct from 20A, 20B, and 20C with no WciG-mediated O-acetylation and WhaF-mediated branching α-Gal*p* residue (Fig. S2B). Thus, PATH4317 represents a new variant within serogroup 20, provisionally named “20X1” (Table S3). We hypothesize that PATH4317 may produce a novel capsule PS and potentially could be classified as a new serotype (Fig. S2B). Altogether, the limited epidemiological data on serogroup 20 isolates suggest that serotype 20C may have a low global prevalence. However, there is evidence of other novel serogroup 20 variants circulating in other parts of the world, thus serogroup 20 isolates should be carefully investigated in future studies.

## DISCUSSION

Herein, we characterize a newly discovered serotype, 20C, within serogroup 20, which was described as a 20B variant (32). Serotype 20C is genetically very similar to serotype 20B but differs in having an inactive *cps*-encoded O-acetyltransferase, WciG. Genetic analysis of multiple 20C *cps* loci identified variable translation defects (truncations) in the WciG, rendering it non-functional. As a result, serotype 20C produces a capsule PS distinct from serotype 20B, and inevitably from 20A. The serotype 20C capsule PS is identical to that of serotype 20B except for the absence of WciG-mediated O*-*acetyl group at tGal*f* (Fig. 2C). Recombinant deletion of *wciG* in a 20B strain resulted in the expression of 20C, confirming the role of the naturally occurring truncations as a result of nonsense *wciG* mutations in the 20C *cps* locus. Thus, loss of *wciG* function appears to be the genetic hallmark of the newly identified serotype 20C.

O-acetyltransferases are known to modify different bacterial surface glycans, such as O-antigen (38), peptidoglycan (39), and capsule PS (40, 41). The resulting acetyl groups are often the dominant targets of serotyping antibodies (41), innate immune molecules (42, 43), and vaccines (44). The *cps* loci of many pneumococcal serogroups/serotypes harbor one or more O-acetyltransferase genes, and some have non-functional relics (45). For instance, serogroup 33 has three different functional O-acetyltransferases, wherein, 33A has both WciG and WcjE, 33F/B/D/E has only WciG, and 33C has only WcyO (7). Akin to the 20B/20C capsule types, multiple cases of pneumococcal syntenic pairs differ according to the presence/absence of WciG-mediated O-acetylation, like, serotype 35C and 42 (46), serotypes 35B and 35D (47). Thus, the functionality of O-acetyltransferases is often a critical distinguishing feature between related serotypes, and the less explored *wciG* functionality appears to be a significant mechanism of increasing capsule diversity among pneumococci.

Accurate typing of serogroup 20 subtypes remains an ongoing challenge (20). Despite distinct biochemical and genetic features, serotype 20C could not be serologically distinguished from serotypes 20A and 20B with currently available serotyping antibodies, making it difficult to estimate the true prevalence of 20C among global populations. Considering the previously noted serologic heterogeneity among serotype 20 strains in inhibiting opsonization (19), we could distinguish 20B and 20C from 20A in an inhibition ELISA, but not 20B from 20C. Given the limited access to assays and/or reagents specific enough to distinguish serogroup 20 subtypes, efforts should develop a type-specific serologic reagent targeting O-acetyl groups or terminal Glc*p* residues to identify these capsule types in conventional serological assays.

Our analysis of 55 “serogroup 20” isolates (20 from the UAB bacterial repository and 35 from GPS) revealed only two 20C isolates obtained from a single continent (Asia). *In silico* analysis of pneumococcal genomes identified unexpectedly multiple serotype 20A isolates recovered from children and adults in various countries (Table S2). However, our limited data, including data from the US IPD isolates (unpublished communication from ABCs), and other studies (16), suggests that 20B seems to be the more prevalent subtype amongst serogroup 20 isolates, with 20A and 20C constituting a smaller proportion. Further evaluation of additional serogroup 20 isolates is necessary to confirm widespread 20C transmission. Nonetheless, existing immunization efforts with 20A PS may be sufficient to manage 20B and 20C infections, as supported by our OPK results.

Regarding the evolutionary origin of serotype 20C, it appears that 20C strains arise from 20B precursors by inactivation of WciG through incremental accumulation of polymorphisms in *wciG*, as opposed to the horizontal gene transfer. Regardless, there is no evidence for clonal propagation of serotype 20C. Furthermore, in the absence of type-specific serological tools, sequencing of *cps* genes, *wciG,* and *whaF*, stands out as the most practical method for surveillance of serogroup 20 subtypes. Interestingly, while studying the genome sequences of new 20C isolates, we discovered a new variant, PATH4317, provisionally named “20X1” which appears to have both *wciG* and *whaF* genes inactive, potentially indicating it to be a new serotype.

Additionally, 20A/B/C capsule types carry a WcjE-mediated O-acetyl group on the Gal*f* residue of the PS backbone that can be inactivated without disrupting the capsule biosynthesis process, as seen with the serotype 9A that can be derived from 9V through the WcjE inactivation (48). Indeed, *in silico* analysis identified 24 bp deletion in the C-terminus of the *wcjE* gene in several 20A *cps* loci (Table S2), and its impact on capsule PS O-acetylation merits investigation. Thus, we predict that within serogroup 20, three *cps* genes—*whaF*, *wciG*, and *wcjE*, can be independently inactivated, and genome sequencing might identify up to eight distinct unrecognized variants (Table S3). Our experience with the serogroup 20 *cps* loci sequences further reinforces the need for serotyping and bioinformatic surveillance tools to inspect all the *cps* genes for mutations that may lead to enzyme inactivation, thereby enabling precise monitoring of emerging capsule variants.

In view of no detectible serologic difference between 20B and 20C, it is unclear if WciG inactivation in 20C would confer unique biologic properties, as observed with other capsule types (49). For instance, 35D is identical to 35B except for its inactive *wciG* (47), and interestingly, 35B reacts with an innate immune factor ficolin-2, unlike 35D, and 35B causes more IPD among older adults than in children (49). Previously, we demonstrated that WciG-mediated O-acetylation is important for producing protective capsules, as *wciG*-deficient variants of serotypes 33A and 33F displayed increased cell wall accessibility, increased nonspecific opsonophagocytic killing, enhanced biofilm formation, and increased adhesion to nasopharyngeal cells (50). Therefore, it would be interesting to compare the biological properties of 20B and 20C in future studies.

## MATERIALS AND METHODS

### Bacterial strains and cultivation

The pneumococcal strains ATCC6320 (serotype 20A) and CDC5931-06 (serotype 20B) were previously described (20). Strain ATCC6320 was obtained from the American Type Culture Collection and clinical isolate CDC5931-06 was obtained from the Centers for Disease Control and Prevention (Atlanta, GA). SPEC6B was derived from a serotype 6B clinical isolate as described previously (51). MNK0184 and MNK0952 are the clinical isolates recovered from patients in South Korea. Other serogroup 20 strains tested in this study are listed in the supplemental table (Table S1). All pneumococcal strains were cultured on blood agar plates with 5% sheep blood (Remel Laboratories, Lenexa, KS). After overnight incubation at 37°C with 5% CO_2_, isolated colonies were inoculated into Todd-Hewitt broth with 5% yeast extract (THY) and grown to mid-log density (optical density at 600 nm [OD_600_] of 0.5) for serological, genetic, and biochemical investigations.

### Construction of pneumococcal mutant strain JY21

Mutant strain JY21 was constructed by recombinant deletion of *wciG* in the *cps* locus of CDC5931-06 using the SJC strategy (7, 34). Briefly, ∼1 kb DNA sequences in each of the upstream and downstream regions flanking *wciG* were PCR-amplified from CDC5931-06 genomic DNA. SJC fragment was PCR amplified from YNL001 genomic DNA. YNL001 is a *wciZ* defective pneumococcal 15B strain, derived from BLS141 by replacing the *wciZ* gene with the SJC (8, 34, 41). The fragments were assembled in a single DNA construct by an overlap extension PCR (52). PCR amplicons were transformed into CDC5931-06, and transformants were selected on THY agar with 300 µg/mL of kanamycin (Kan). Genomic recombination was confirmed by Sanger sequencing performed at the Heflin Center Genomics Core Lab at the UAB. The primers used to create JY21 are listed in Table S4.

### Capsule PS purification

Capsule PS was purified from strains CDC5931-06, MNK0184, MNK0952, and JY21 by ion exchange chromatography as described previously (6, 8). Briefly, a single bacterial colony was cultured in 10 mL THY broth and expanded in 1 L of chemically defined medium supplemented with choline chloride (1 g/L), sodium bicarbonate (2.5 g/L), and cysteine HCl (0.73 g/L) (53). Following overnight incubation at 37°C, the bacteria were centrifuged (15,344 × *g*, 30 min, 4°C), washed, and resuspended in 0.9% NaCl to a final volume of 20 mL. The pH was adjusted to 7 with 3 N NaOH, and the suspension was treated with 100 µL of 10% sodium deoxycholate, 200 µL of mutanolysin (10 U/µL), and then incubated at 37°C for 48 to 72 h. The lysate was centrifuged, dialyzed, and applied to a DEAE Sepharose anion-exchange column (GE Healthcare, Uppsala, Sweden). The bound material was eluted with a NaCl gradient from 0 to 400 mM in 5 mM Tris-HCl. Each fraction was tested for OD_260_, OD_280_, teichoic acid, and the presence of capsule PS with an anthrone assay and inhibition-type enzyme-linked immunosorbent assay (iELISA) as described below.

### Detection of purified capsule PS: anthrone reaction and inhibition-type ELISA

Elution fractions from the ion exchange chromatography were analyzed using a modified anthrone assay (35, 54) to detect carbohydrates. Briefly, 50 µL of the fraction was mixed with 50 µL of 0.02% (wt/vol) anthrone (Sigma) in sulfuric acid. The mixture was boiled for 10 min in a water bath and then cooled to room temperature (RT). The absorbance was measured at OD_630_. Fractions containing carbohydrates develop light to dark greenish color.

Fractions that showed positive reactivity in anthrone assay were further tested for the presence of specific capsule PS and teichoic acid with iELISA as described previously (6). For capsule PS detection, the ELISA plates (Corning Costar Corp., Acton, MA) were coated with 1 µg/mL of commercially available 20A capsular PS (ATCC, 209-X, ID 950121). Fifty microliter portions of the samples containing PS were added to the wells along with 50 µL of type-20 specific Mab, Hyp20G5, at 1:100 dilutions. Teichoic acid was detected by using anti-phosphocholine Mab, HPCG2b, at 1:100 dilutions (50, 55). Bound antibodies were detected with alkaline phosphatase-conjugated goat anti-mouse Ig (Southern Biotech) at 1:3,000 dilutions. The amount of enzyme immobilized to Hyp20G5 or HPCG2b was detected by incubating in paranitrophenyl phosphate substrate (Sigma) in diethanolamine buffer, and the absorbance was read at OD_405_. The fractions with a high polysaccharide concentration, and low teichoic acid concentration, were pooled, dialyzed, and lyophilized.

### Flow cytometric serotyping assay

The phenotypic expression of the capsule PS on the bacterial surface was detected by flow cytometry as described previously (47, 56) using polyclonal rabbit antisera—type 20 antiserum and factor sera 20b, obtained from the Statens Serum Institut (SSI, Copenhagen, Denmark), or using our in-house type-20 specific Mab, Hyp20G5. The MAb was produced as described previously (57). Briefly, frozen bacterial stocks were thawed, washed, and incubated in FCSA buffer (phosphate-buffered saline, 3% fetal bovine serum, 0.1% NaN_3_) containing appropriate dilutions of polyclonal or Mab antisera for 30 min at 4°C. After washing, bound immunoglobulin (Ig) was stained with phycoerythrin-labeled anti-rabbit or anti-mouse Ig antibody (Southern Biotech, Birmingham, AL), and detected by flow cytometry using BD Accuri C6 Plus (BD Biosciences, Franklin Lakes, USA) and FCS Express software (Pasadena, USA).

### Monosaccharide composition analysis

The monosaccharide composition of purified capsule PS from strains CDC5931-06, MNK0184, and JY21 was determined by acid hydrolysis followed by high-performance anion-exchange chromatography-pulsed amperometric detection (HPAEC-PAD) (58). PS samples and monosaccharide standards—Glucose (Glc), Galactose (Gal), and N-acetylglucosamine (GlcNAc) were hydrolyzed by incubating with 2 N trifluoroacetic acid at 121°C on a heat block for 1 h. The resulting hydrolysates were subjected to vacuum centrifugation at 45°C to evaporate solvents followed by reconstitution in milliQ water. Monosaccharides were identified by HPAEC-PAD using a Dionex ICS-6000 (Thermo Fisher Scientific) on a Carbopac PA20 column (Thermo Fisher Scientific). Quantitation of monosaccharides derived from PS samples was carried out by creating a standard curve with hydrolyzed monosaccharide standards, ranging from 0.625 µM to 10 µM.

### NMR spectroscopy

Approximately, 5 mg of purified capsule PS samples were dissolved in 0.6 mL of 99.99% D_2_O (Cambridge Isotope Laboratories). ^1^H NMR data were collected at 35°C on Bruker Avance III-HD (^1^H, 600 MHz) spectrometers equipped with cryogenic triple-resonance probe. ^1^H NMR spectrum was obtained by water suppression using a presaturation pulse sequence (zgpr). Complete assignments of ^1^H and ^13^C signals were achieved by two-dimensional ^1^H-^1^H, ^1^H-^31^P, and ^1^H-^13^C NMR correlation experiments recorded at 50°C. NMR data were processed and analyzed with Bruker TopSpin 3.6.2 software. HDO signal was used as a reference.

### Molecular modeling

Models were built with CarbBuilder version 2.1.46 (59), using conformations of the glycosidic linkages obtained from the global minimum in corresponding calculated disaccharide potential of mean force (calculated with the metadynamics routine incorporated into NAMD [60], with the ϕ, ψ glycosidic linkage torsion angles used as collective variables).

### Opsonophagocytosis assay

To investigate whether 20A PS in PPSV23 elicits cross-opsonizing antibodies to serotype 20B and 20C, we adapted a well-characterized UAB opsonophagocytosis assay (OPA) (51) (and described in detail at https://www.vaccine.uab.edu/uploads/mdocs/UAB-MOPA.pdf). OPA was performed with four anonymous post-PPSV23 immunized human serum samples and 007sp, a reference serum from FDA prepared by pooling sera from vaccinated adults (36, 37), using ATCC6320, CDC5931-06, and MNK0184 as targets. Briefly, 10 µL of bacterial suspension (∼10^5^ CFU/mL) and 20 µL of serially diluted antiserum were incubated in a microtiter plate for 30 min at RT with shaking (700 rpm). Next, 10 µL of baby rabbit complement (PelFreeze Biologicals, Rogers, AK) and 40 µL of differentiated HL60 cells (10^7^ cells/mL) were added to each well, and plates were incubated with shaking (700 rpm) for 45 min at 37°C with 5% CO_2_. Ten microliters from each well were spotted on THY agar plates, and the bacterial colonies were counted after overnight incubation. Opsonic indices were determined as the serum dilution that kills 50% of bacteria by linear interpolation.

### Genome sequence analysis

Genomic DNA was extracted from the pneumococcal strains listed in Table S1 using a Monarch Genomic DNA purification kit (New England Biolabs). *wciG* gene was PCR amplified using primers listed in Table S4 and subjected to Sanger sequencing, which was performed by the Heflin Center Genomics Core Lab at the UAB. For strains with available WGS data (Table S2), *cps* loci were extracted and analyzed by comparing with the reference *cps* loci sequences—20A *cps* locus (JQ653094) and 20B *cps* locus (JQ653093). Nucleotide and amino acid sequences were compared, translated, and analyzed by Geneious prime v2020. Multiple Alignment using Fast Fourier Transform was run with a scoring matrix of 200 PAM/K of 2 and a gap open penalty of 1.5.

## SUPPLEMENTAL MATERIAL

Figures S1 and S2; Tables S1 to S4 spectrum.02443-24-s0001.pdf Fig. S1: Heteronuclear multiple quantum coherence (1H-13C HMQC) NMR spectrum of MNK0184. Fig. S2: New genetic variant of serogroup 20. Table S1: List of pneumococcal strains evaluated in this study. Table S2: List of serogroup 20 isolates from the GPS database. Table S3: Predicted novel genetic variants of serogroup 20. Table S4: Primers used in this study.

ASM does not own the copyrights to Supplemental Material that may be linked to, or accessed through, an article. The authors have granted ASM a non-exclusive, world-wide license to publish the Supplemental Material files. Please contact the corresponding author directly for reuse.
