# Supplementary material for: New pneumococcal serotype 20C is a WciG O-acetyltransferase deficient variant of canonical serotype 20B
Source: Microbiol Spectr. 2024 Nov 29;13(1):e02443-24. doi: 10.1128/spectrum.02443-24 (PMC11705870; doi:10.1128/spectrum.02443-24)
Supplement: Figures S1 and S2; Tables S1 to S4 — Fig. S1: Heteronuclear multiple quantum coherence (1H-13C HMQC) NMR spectrum of MNK0184. Fig. S2: New genetic variant of serogroup 20. Table S1: List of pneumococcal strains evaluated in this study. Table S2: List of serogroup 20 isolates from the GPS database. Table S3: Predicted novel genetic variants of serogroup 20. Table S4: Primers used in this study. [file spectrum.02443-24-s0001.pdf]

Supplementary material for:

New pneumococcal serotype 20C is a WciG O-acetyltransferase deficient variant of canonical serotype 20B

Jigui Yu[1], Neil Ravenscroft[2], Peter Davey[3], Roshan Liyanage[3], Oliver Lorenz[4], Michelle M. Kuttel[5], Stephanie W. Lo[4,6], Feroze A. Ganaie[1,#], Moon H. Nahm[1,#]

[1]Department of Medicine, Division of Pulmonary/Allergy/Critical care, University of Alabama at Birmingham, Birmingham, AL, USA

[2]Department of Chemistry, University of Cape Town, Rondebosch, South Africa

[3]Vaxcyte, San Carlos, CA, USA

[4]Parasites and Microbes, Wellcome Sanger Institute, Hinxton, Cambridge, UK

[5]Department of Computer Science, University of Cape Town, Rondebosch, South Africa

[6]Milner Centre for Evolution, Department of Life Sciences, University of Bath, Bath, UK

Running title: Discovery of a new pneumococcal serotype, 20C

[#]Address correspondence to Moon H. Nahm, mnahm@uabmc.edu

[#]Alternate correspondence to Feroze A. Ganaie, fganaie@uabmc.edu

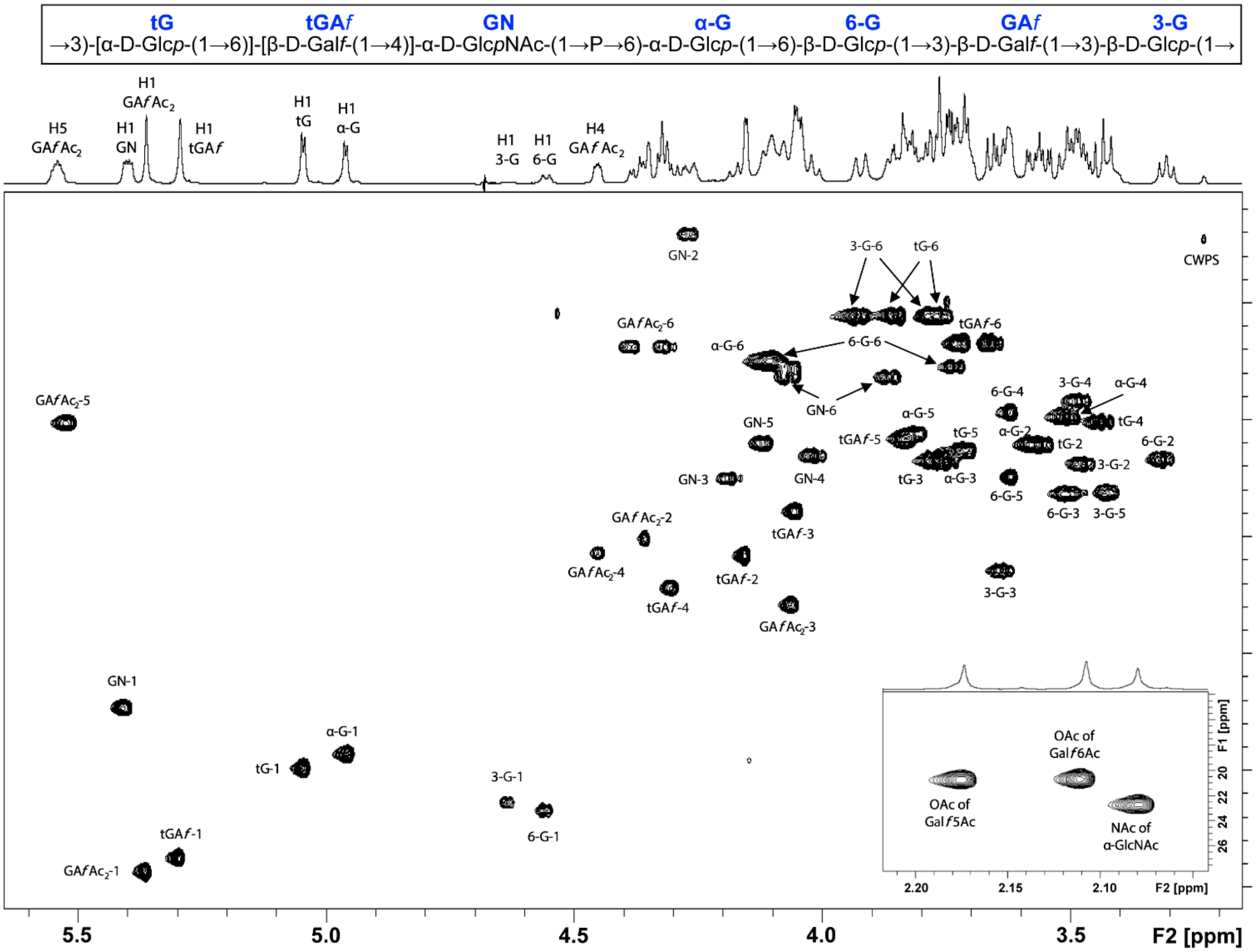

Fig. S1. Heteronuclear multiple quantum coherence (^{1}H-^{13}C HMQC) NMR spectrum of MNK0184. The inset shows the methyl region. Recorded on a Bruker Avance III 600 MHz NMR spectrometer equipped with cryogenic triple-resonance probe at 50°C with 25% NUS, 4k × 256 data points and J = 145 Hz (for directly attached ^{1}H-^{13}C correlations), Assignments established using 2D ^{1}H-^{1}H, ^{1}H-^{31}P and ^{1}H-^{13}C NMR correlation experiments. The peak labels indicate the sugar residue (shown on top of the 20C repeat unit structure in blue letters) and the number denotes the carbon position on each unit. MNK0184 and MNK0952 produced identical 2D NMR data; however, for clarity, we are showing only the MNK0184 data.

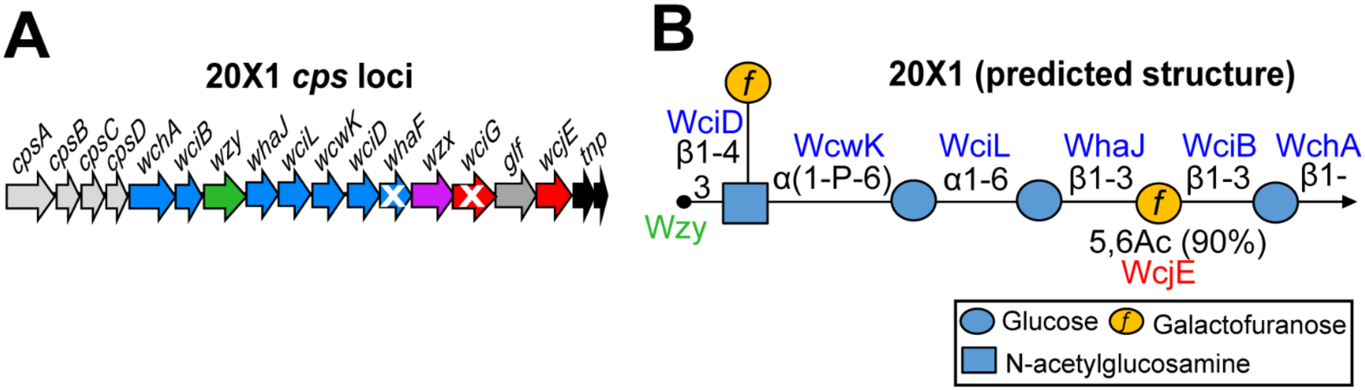

Fig. S2. New genetic variant of serogroup 20. (A), *cps* locus of PATH4317, which may express a novel capsule type (serotype), provisionally named as "20X1". Highly conserved regulatory genes (light gray arrows), genes encoding glycosyltransferases (blue arrows), Wzy polymerase (green arrow), Wzx flippase (pink arrow), O-acetyltransferases (red arrows), carbohydrate synthetase (dark grey arrow), transposable elements (black arrows) are labelled at the top of 20X1 *cps* locus. White color cross on *whaF* and *wciG* gene arrows indicates that these genes are defective. *cps*, capsule synthesis locus. **(B)**, Predicted structure of 20X1 PS repeat unit based on the *cps* gene content. *cps*-encoded glycosyltransferase, O-acetyltransferases, and polymerase are listed in blue, red and green text, respectively, on top of their putatively assigned linkages. PS, polysaccharide.

Table S1. List of pneumococcal strains evaluated in this study

Strain name	Serotype	Type	Source	wciG	Accession
ATCC6320	20A	n/a	n/a	Intact	JQ653094
CDC5931-06	20B	IPD	n/a	intact	JQ653093
CDC3014-06	20B	IPD	n/a	intact	n/a
CDC3049-06	20	IPD	n/a	intact	n/a
CDC3050-06	20	IPD	n/a	intact	n/a
CDC3916-06	20	IPD	n/a	intact	n/a
CDC3917-06	20	IPD	n/a	intact	n/a
CDC3947-06	20	IPD	n/a	intact	n/a
CDC4579-06	20B	IPD	n/a	intact	n/a
CDC5752-06	20	IPD	n/a	intact	n/a
CDC6666-06	20	IPD	n/a	intact	n/a
CDC6684-05	20	IPD	n/a	intact	n/a
CDC7069-05	20	IPD	n/a	intact	n/a
MNK0025	20B	carriage	Older adult	intact	n/a
MNK0027	20B	IPD	Older adult	intact	n/a
MNK0362	20	carriage	Older adult	intact	n/a
MNK0642	20B	carriage	Older adult	intact	n/a
MNK0884	20	carriage	Older adult	intact	n/a
MNK1056	20B	n/a	n/a	intact	n/a
MNY339	20	n/a	n/a	intact	n/a
MNK0184	20C	carriage	Older adult	Defective	PQ205320
MNK0952	20C	n/a	Older adult	Defective	PQ205321

This list includes pneumococcal strains from the UAB bacterial repository only. n/a, information not available.

Table S2. List of serogroup 20 isolates from the GPS database

Public name	In-silico serotype	Country	GPSC	Manifestation	Source	*wciG*	*whaF*	*wcjE*	Accession no.
GPS_ZA_SP74[$]	20A	South Africa	22	Carriage	Children	intact	714 bp deletion[#]	24 bp deletion*	ERS638650
GPS_ZA_SP82[$]	20A	South Africa	22	Carriage	Children	intact	714 bp deletion[#]	24 bp deletion*	ERS638714
GPS_ZA_SP177[$]	20A	South Africa	22	Carriage	Children	intact	714 bp deletion[#]	24 bp deletion*	ERS638904
GPS_GM_1486	20A	The Gambia	61	Carriage	Children	intact	714 bp deletion[#]	24 bp deletion*	ERS654555
GPS_GM_2545	20A	The Gambia	61	Carriage	Adult	intact	714 bp deletion[#]	24 bp deletion*	ERS811890
GPS_GM_2428	20A	The Gambia	61	Carriage	Children	intact	714 bp deletion[#]	24 bp deletion*	ERS811807
GPS_GM_2842	20A	The Gambia	61	Carriage	Children	intact	714 bp deletion[#]	24 bp deletion*	ERS1222389
GPS_GM_2751	20A	The Gambia	61	Carriage	Children	intact	714 bp deletion[#]	24 bp deletion*	ERS1222298
GPS_GM_2755	20A	The Gambia	61	Carriage	Children	intact	714 bp deletion[#]	24 bp deletion*	ERS1222302
GPS_GM_2906	20A	The Gambia	61	Carriage	Children	intact	714 bp deletion[#]	24 bp deletion*	ERS1813316
GPS_GM_3119	20A	The Gambia	61	Carriage	Children	intact	714 bp deletion[#]	24 bp deletion*	ERS1813588
GPS_GM_2676_R1	20A	The Gambia	61	Carriage	Children	intact	714 bp deletion[#]	24 bp deletion*	ERS1813544
GPS_GM_3090	20A	The Gambia	61	Carriage	Children	intact	714 bp deletion[#]	24 bp deletion*	ERS1813552
GPS_GM_3030	20A	The Gambia	61	Carriage	Children	intact	714 bp deletion[#]	24 bp deletion*	ERS1813446
GPS_GM_3300	20A	The Gambia	61	IPD	Children	intact	714 bp deletion[#]	24 bp deletion*	ERS1574418
GPS_GM_3319	20A	The Gambia	61	Carriage	Adult	intact	714 bp deletion[#]	24 bp deletion*	ERS1574439
GPS_GM_3234	20A	The Gambia	61	Carriage	Children	intact	714 bp deletion[#]	24 bp deletion*	ERS1574339
GPS_PK_740131	20A	Pakistan	61	Non-invasive	Elderly	intact	714 bp deletion[#]	24 bp deletion*	ERS4307791
GPS_GM_2594	20A	The Gambia	91	Carriage	Adult	intact	714 bp deletion[#]	24 bp deletion*	ERS811970
GPS_MW_18645	20A	Malawi	184	Carriage	Unknown	intact	714 bp deletion[#]	intact	ERS625655
GPS_MW_19043	20A	Malawi	184	Carriage	Unknown	intact	714 bp deletion[#]	intact	ERS625976
GPS_PK_098	20A	Pakistan	184	IPD	Children	intact	714 bp deletion[#]	intact	ERS1151466
GPS_NP_8051	20A	Nepal	184	Carriage	Children	intact	714 bp deletion[#]	intact	ERS2994631
GPS_IN_P04	20B	India	43	IPD	Children	intact	intact	24 bp deletion*	ERS1091663
GPS_IN_P47	20B	India	43	IPD	Children	intact	intact	24 bp deletion*	ERS1206291
GPS_NP_8091	20B	Nepal	43	Carriage	Children	intact	intact	Intact	ERS2994650
GPS_NP_8133	20B	Nepal	43	Carriage	Children	intact	intact	Intact	ERS2994701
GPS_PL_1345_06	20B	Poland	61	IPD	Elderly	intact	intact	24 bp deletion*	ERS628974
GPS_BR_0711_09	20B	Brazil	124	IPD	Adult	intact	intact	Intact	ERS1299735
GPS_IL_27574	20B	Israel	124	IPD	Elderly	intact	intact	intact	ERS1699601

GPS_IL_8634	20B	Israel	124	IPD	Elderly	intact	intact	intact	ERS1699669
GPS_BR_0641_09_R1	20B	Brazil	124	IPD	Children	intact	intact	intact	ERS2553124
GPS_TH_BK_0346	20B	Thailand	682	IPD	Elderly	intact	intact	intact	ERS634613
MY_UMSP_30	20B	Malaysia	682	IPD	Elderly	intact	intact	intact	ERS762330
GPS_US_PATH4317	20X1	Ethiopia	667	Carriage	Unknown	163 bp insertion[¶]	714 bp deletion[#]	24 bp deletion*	ERS1021286

$Full public name of strains— GPS_ZA_SP74 (GPS_ZA_CARRIAGE_SP74), GPS_ZA_SP82 (GPS_ZA_CARRIAGE_SP82), and GPS_ZA_SP177 (GPS_ZA_CARRIAGE_SP177). [¶]163 bp insertion between nucleotides 301 and 465, resulting in the premature translation termination of WciG at amino acid residue 107. [#]714 bp deletion in the middle of *whaF* gene, resulting in the loss of 238 amino acids between residues 42 and 281. *24 bp deletion at the 3′ end of *wcjE* gene

Table S3. Predicted novel genetic variants of serogroup 20

**Serogroup 20 variants**	***cps* genes**		
	whaF	***wciG***	***wcjE***
20A	Defective	Intact	Intact
20B	Intact	Intact	Intact
20C	Intact	Defective	Intact
20X1	Defective	Defective	Intact
20X2	Intact	Defective	Defective
20X3	Intact	Intact	Defective
20X4	Defective	Intact	Defective
20X5	Defective	Defective	Defective

Table S4. Primers used in this study

Primer name	Sequence	Target region
51509	CGAAATATCACAGTCAAACTCGTTGGTGTC	5´ flanking region
31274	GCATTATCCGGTACCTCTCTCTAGTTTTTCCTAATAATTTGTTTTAATTCTTTCACATCTACCAC	
51510	TTATTAGGAAAAACTAGAGAGAGGTACCGGATAATGCTGAAAACTCCTTG	Sweet Janus
31275	CTTGTTCTCCTTTGAGACTCGAGCCTTTCCTTATGCTTTTGG	
51511	GGCTCGAGTCTCAAAGGAGAACAAGATGTACGATTATCTTATTGTCG	3´ flanking region
31276	AGCTTCCTCTTGGTACTTAGCAAACATAGCAT	
51429	GTTCTAGCATTTGCAGCGCTTGG	5´ *wciG*
31189	GAAGGTATTCATATTGAAAGGTAGATTATAGAGGCTACCCT	3´ *wciG*
